# Genetic resources and genomics for adaptation of livestock to climate change

**DOI:** 10.3389/fgene.2014.00461

**Published:** 2015-01-19

**Authors:** Paul J. Boettcher, Irene Hoffmann, Roswitha Baumung, Adam G. Drucker, Concepta McManus, Peer Berg, Alessandra Stella, Linn B. Nilsen, Dominic Moran, Michel Naves, Mary C. Thompson

**Affiliations:** ^1^Animal Production and Health Division, Food and Agriculture Organization of the United NationsRome, Italy; ^2^In Situ and On Farm Conservation, Bioversity InternationalMaccarese, Italy; ^3^INCT Pecuaria, Universidade de BrasíliaBrasilia, Brazil; ^4^NordGen Farm Animals, Nordic Genetic Resource CenterÅs, Norway; ^5^Parco Tecnologico PadanoLodi, Italy; ^6^Institute of Agricultural Biology and Biotechnology, National Research CouncilMilan, Italy; ^7^Plant Production and Protection Division, Food and Agriculture Organization of the United NationsRome, Italy; ^8^NADEL – Center for Development and Cooperation, Swiss Federal Institute of Technology in ZürichZürich, Switzerland; ^9^Sustainable Rural Systems, Scotland's Rural CollegeEdinburgh, UK; ^10^UR143, Unité de Recherches Zootechniques, Institut National de la Recherche AgronomiquePetit-Bourg, Guadeloupe, France; ^11^Basque Center for Climate ChangeBilbao, Spain

**Keywords:** animal genetic resources, adaptation, biological, genetic diversity, livestock genomics, climate change

**A commentary on**

**Global plan of action for animal genetic resources and the interlaken declaration**

by FAO. (2007). Rome. ISBN: 978-92-5-105848-0

## Introduction

Animal genetic resources (AnGR) are critical for global food security and livelihoods. Livestock products have high densities of energy, protein, and other critical nutrients, which are particularly beneficial for infants and expectant mothers. Around a billion people rely directly on livestock for their livelihoods, many of which are among the rural poor (FAO, 2009). Demand for animal products is foreseen to increase significantly in the future while competition for resources will intensify, dictating that livestock systems must increase both productivity and efficiency. Maintaining sufficient diversity of AnGR is necessary to ensure adaptation potential in times of uncertainty. In the future, climate change is expected to be a major force testing resilience of global food production systems (Thornton et al., 2009; Renaudeau et al., 2012). Ensuring that livestock systems remain productive and efficient while maintaining their flexibility will be a major challenge.

Adaptation to climate change is unlikely to be achieved with a single strategy (Hoffmann, 2010). Clearly, modifications will be needed in animals' housing, reproduction, nutrition, and health care. Genetic changes in the animals (both within and across species) will also play a role. Preparation for these transformations will require a significant research commitment and genomics will play a role in the genetic measures taken for adaptation of livestock to climate change.

## Information needs

The first step in this process will be gathering relevant information. There is currently a shortage of specific knowledge explaining why certain AnGR are adapted to a given environment and in which other environments they can survive and flourish. This deficit calls for greater effort in characterization of AnGR, including their production environments, by using the most modern tools available. To date, many livestock breeds have been genetically characterized (e.g., Groeneveld et al., 2010), but the value of these data for study of adaptation is questionable. For climate change adaptation, the breeds of greatest interest may be those reared today in harsh environments. Past studies have mostly addressed breeds from developed countries, where climate-control is widely practiced. The study of adaptation implies the use of a “landscape approach,” with detailed information describing the production system (e.g., FAO, 2012), including socio-economic information (e.g., Drucker, 2010) and indigenous knowledge about management of the breed in its environment as well as geographic coordinates to incorporate climatic data and soil, vegetation, and water resources. Collection of such detailed complementary data is a relatively recent trend. Past studies have emphasized pure breeds, whereas crossbreeding can be a valuable strategy for achieving increased productivity and adaptability, so those populations also require characterization. Finally, many studies have assayed only small numbers of selectively-neutral markers. The rapid development of genomic tools now allows analysis of functional genomic regions with potential associations with adaptation (e.g., Qian et al., 2013).

## Selection objectives and strategies

A second research need is the elaboration of the genetic objective for adaptation. Increasing productivity and efficiency will be fundamental, but maintenance of genetic diversity will also be of importance. Having diverse AnGR will allow for more opportunities to match breeds to a changing climate or to replace populations hit by severe climatic events such as droughts and floods. Within breed, broad genetic diversity will clearly allow for greater opportunities for selection for adaptation, but there is evidence from wild populations that increased genetic diversity is also selectively advantageous on the individual level (Fourcada and Hoffman, 2014). Directional selection for adaptive traits will likely accompany maintenance of diversity, but questions remain about indicators of adaptation and resilience and hence the breeding goal. An obvious option is to breed for traits associated with superior productivity and resilience in conditions expected to be prevalent as a result of climate change, such as heat and drought tolerance and resistance to certain diseases. However, care must be taken when defining such traits. In dairy cattle, the ability to maintain high milk production with increasing ambient temperatures seems like a logical definition of heat tolerance, but research at the physiological level has shown that such cattle direct their energy toward milk production, making them vulnerable to extremely high temperatures (Dikmen et al., 2012). An alternative to breeding for specific traits is to target general robustness; the ability of animals to adjust to a range of environmental conditions. The Brown Swiss dairy cow, developed under comparatively cool, but rugged conditions in the Alps, seems to show greater heat tolerance than the Holstein (Correa-Calderon et al., 2005), which originated in more temperate lowland regions. The Domestic Animal Diversity Information system of FAO (http://dad.fao.org) lists numerous breeds, particularly from mountainous and arid areas, that are adapted to extreme ranges in temperature and such breeds may merit further research (Hoffmann, 2013). Table 1 has the numbers of local breeds by region and species for the major livestock species, as recorded in DAD-IS. Within-breed selection for robustness would likely require the development of an index involving multiple traits.

**Table 1:** **Numbers of local^a^ breeds recorded per species and region for the six main livestock species^b^ for food and agriculture, according to the Domestic Animal Diversity Information System (DAD-IS) of FAO (http://dad.fao.org – November 2014)**.

**Species**	**Africa**	**Asia**	**Europe and the Caucasus**	**Latin America and the Caribbean**	**Near and Middle East**	**North America**	**Southwest Pacific**	**World**
Cattle	176	241	369	141	43	17	32	1019
Chicken	129	305	912	88	35	15	30	1514
Sheep	117	262	613	51	53	21	38	1155
Horse	40	138	371	84	14	22	25	694
Goat	96	183	218	28	34	6	11	576
Pig	53	214	188	60	1	12	15	543
Duck	15	92	107	22	4	1	12	253
Others	171	328	568	123	67	37	27	1321
Total	797	1763	3346	597	251	131	190	7075

a *Local breed, a breed reported in only one country, according to DAD-IS*.

b *Main species determined based on the number of breeds*.

A third category of research involves the genetic strategy for adaptation. Options comprise purebreeding, crossbreeding (including introgression), and breed or species substitution. Among the key influential factors is the expected rate of climate change and the speed with which genetic change can realistically occur with the various strategies. Substitution and crossbreeding expedite genetic change, but their implementation may be more complex than purebreeding and thus involve additional research needs (e.g., on genotype-by-environment interaction).

## Tools for the genetics of adaptation

A final research topic will be the development of tools required for the aforementioned topics. Genomics will surely play a role in all three of these areas, as well as in implementation of the results obtained. Increased characterization with high-throughput single nucleotide polymorphism (SNP) assays or genome sequencing will be necessary for unraveling the physiological basis for adaptation. Species-wide HapMap studies (e.g., Gibbs et al., 2009; Jiang et al., 2014) and multi-species studies (e.g., Stella, 2014) have represented a valuable first step in understanding the genome and its function in adaptation, but must be expanded to more breeds and geographical areas and augmented with more information on production environments. Metagenomics can provide insight regarding the co-adaptation of AnGR with other organisms in their production environments. Genomic selection has the potential to expedite both pure- and crossbreeding programmes for adaptation, assuming phenotypes are available (Hayes et al., 2012); programs for performance recording in developing countries are thus needed. Given the importance of the landscape approach, tools and methods for improved integration of geographical information will be critical. The information gathered in all of these processes will be of little value if they are not properly organized, stored, and disseminated to stakeholders through new and improved databases and information systems. Finally, cooperative efforts between all stakeholders will be needed to achieve the final goal, the optimal utilization of the genetic and genomic resources for the adaptation of livestock to climate change.

### Conflict of interest statement

The authors declare that the research was conducted in the absence of any commercial or financial relationships that could be construed as a potential conflict of interest.
